# The distribution and characteristics of LDL receptor mutations in China: A systematic review

**DOI:** 10.1038/srep17272

**Published:** 2015-11-26

**Authors:** Long Jiang, Li-Yuan Sun, Yan-Fang Dai, Shi-Wei Yang, Feng Zhang, Lu-Ya Wang

**Affiliations:** 1 Beijing Anzhen Hospital, Affiliated to Capital Medical University, Beijing Institute of Heart, Lung and Blood Vessel Diseases. The Key Laboratory of Remodelling-related Cardiovascular Diseases, Ministry of Education, Department of Atherosclerosis, Beijing 100029, China; 2 Beijing Anzhen Hospital, Affiliated to Capital Medical University, Department of Dermatology, Beijing 100029, China; 3 Institute of Genomics, Chinese Academy of Sciences and Key Laboratory of Genome Science and Information, Chinese Academy of Sciences, Beijing 100101, China; 4 National Engineering Laboratory for Druggable Gene and Protein Screening, Northeast Normal University, Changchun 130024, Jilin, China

## Abstract

Familial hypercholesterolemia (FH) is a common and serious dominant genetic disease, and its main pathogenic gene is the low-density lipoprotein receptor (LDLR) gene. This study aimed to perform a systematic review of LDLR mutations in China. Using PubMed, Embase, Wanfang (Chinese), the Chinese National Knowledge Infrastructure (Chinese), and the Chinese Biological and Medical database (Chinese), public data were limited to December 2014. The Medical Subject Headings terms and the following key words were used: “familial hypercholesterolemia”, “Chinese”, “China”, “Hong Kong”, and “Taiwan”. A total of 74 studies including 295 probands with 131 LDLR mutations were identified. Most of the mutations were located in exon 4 of LDLR and approximately 60% of the mutations were missense mutations. Thirty new mutations that were not recorded in the LDLR databases were found. In silico analysis revealed that most of the mutations were pathogenic. The primary LDLR mutations were C308Y, H562Y, and A606T, and all of the mutations had functional significance. Prevalence data suggest that there are nearly 3.8 million FH patients in China, although reported numbers are much smaller, suggesting that FH is widely misunderstood. This systematic review provides information that is specific to China for inclusion in the international FH database.

Familial hypercholesterolemia (FH, OMIM: #143890), which is characterized by tendon xanthoma, severely elevated LDL cholesterol (LDL-C) and premature coronary heart disease (pCHD), is a common and serious dominant genetic disease and has recently become a topic of extensive concern worldwide^1^. LDL-C levels are elevated 2- to 3-fold in heterozygous FH (HeFH) patients, and these patients progress to CHD before age 45 if they are not treated. Homozygous FH (HoFH) patients exhibit 6- to 8-fold increases in plasma LDL-C and a severe phenotype; these patients also develop serious cardiovascular disease before the age of 12.5 years if untreated^2^. Recently, HeFH and HoFH were found to occur in approximately 1/200 to 1/500 of the population and 1/160 000 to 1/300 000 of the population, respectively, which is higher than previously reported values^3^. One Chinese study reported that the prevalence of probable/definite FH was 0.28% (1.4/500) based on the modified Dutch lipid clinic network (DLCN) definition, which is similar to the worldwide prevalence^4^. Hence, there are nearly 36 million potential FH patients in the world, including 3.8 million patients in China. However, current data have shown that FH is underdiagnosed and undertreated in most counties, especially in mainland China, where only ~100 index patients have been reported^5^. Therefore, there is likely a lack of understanding of FH among the general public in mainland China.

FH is a monogenic autosomal dominant disease in which a single causative mutation in the pathogenic gene affects cholesterol metabolism. The major pathogenic genes for FH are low-density lipoprotein receptor (LDLR, MIM 606945), apolipoprotein B (APOB, MIM107730), and proprotein convertase subtilisin/kexin type 9 (PCSK9, MIM 607786)^1^. The most important pathogenic gene is LDLR, and approximately 90% of FH patients have mutations in this gene. The other pathogenic genes have been reported less frequently worldwide, especially in China. Based on the current LOVD databases (www.ucl.ac.uk/ldlr/LOVDv.1.1.0/ and https://grenada.lumc.nl/LOVD2/UCLHeart/home.php?select_db=LDLR), there are more than 1700 mutations in the LDLR gene worldwide, reflecting the extensive genetic heterogeneity of FH patients, especially among different races. Researchers have speculated that the reason for this heterogeneity may be related to genetic drift, the founder effect, or intermarriage between FH patients. Large-scale clinical studies of FH have been conducted in many countries, but fewer data have been reported from China. Our group began to focus on FH in 2003 and has contributed to research on FH in China^6^,^7^,^8^,^9^,^10^. Here, we provide a general overview of causative LDLR mutations in China, including the characteristics of the geographical distribution and phenotypes of patients with LDLR mutations and discussion of whether various other factors, such as the founder effect, are present.

## Results

### Study selection and research status of FH in China

**Figure 1:**
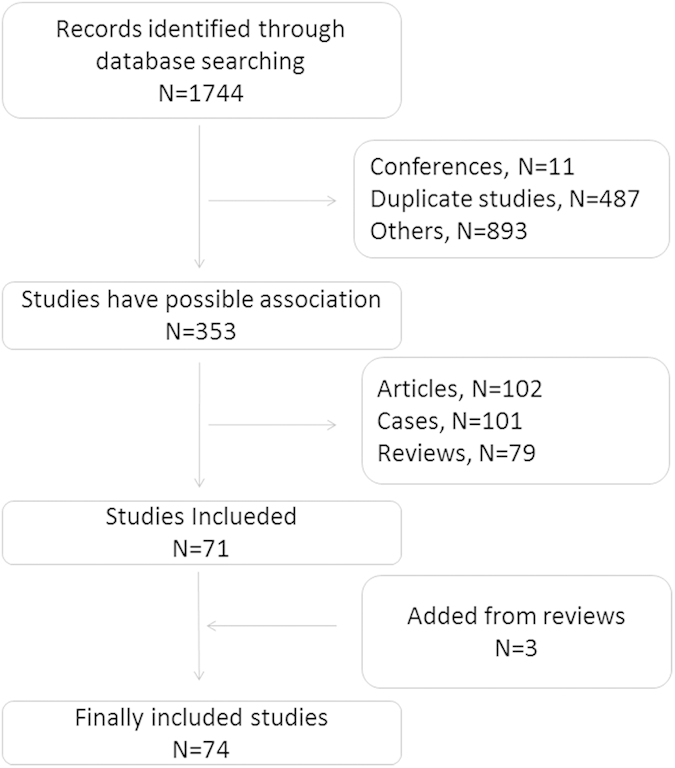
Flow chart of the study selection.

Researchers reported the first Chinese case of FH in 1971^11^. Subsequently, a number of FH patients were reported in Hong Kong, Taiwan Province, and Jiangsu Province, among others. A total of 1744 studies were analyzed for this review. After the removal of conference papers, duplicate articles and other unrelated studies, 353 potentially related studies were included in the analysis. In addition, three further studies were identified by reading reviews. Therefore, a total of 356 studies related to Chinese FH were selected. Of these, 74 studies that recorded mutations in FH were included in this systematic review (Fig. 1). A comprehensive literature analysis showed that the number of published articles on FH has increased each year, especially after 2004; 57.3% of the total articles were published after 2004 (Supplemental Fig. S1 online). However, more than half of the studies were case reports and reviews. In addition, many researchers reported a single pedigree with FH, and fewer than 23.6% of the studies performed functional experiments to investigate causative mutations.

### LDLR mutations in mainland China, Hong Kong and Taiwan Province

#### Overall results

**Figure 2:**
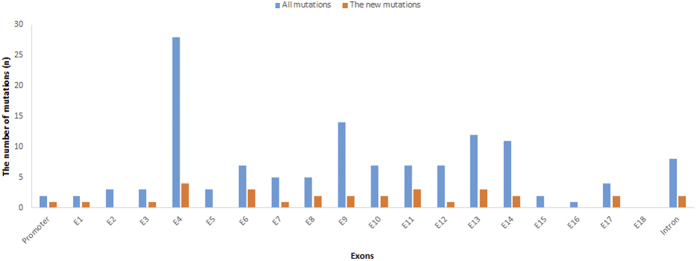
Distribution of Chinese LDLR mutations.

In 1985, Cai *et al.* identified the first five Chinese HoFH patients using a radio-labeled ligand assay, although the technology at that time could not be used to define the site of the mutation^12^. Later, Hobbs and Sun *et al.* identified LDLR mutation sites and their functions using PCR and cDNA cloning technology, respectively, in patients with FH from Jiangsu Province^13^,^14^. In our systematic review, we found 63 related studies that reported Chinese LDLR mutation sites. Thus far, 295 probands, including 131 LDLR mutations, have been reported (Supplemental Table S1 online); the geographical distribution of the characteristics of the LDLR mutations is shown in Supplemental Figure S2 online. The distribution of mutations revealed that most of the mutations were located in exon 4, and the next largest percentages of mutations were located in exons 9, 13, and 14. There was also a high number of intronic mutations, similar to a recently reported study^15^ (Fig. 2). In addition, approximately 60.3% (79/131) of the mutations were missense mutations, 13% (17/131) were nonsense mutations, and 2.3% (3/131) were large fragment deletion mutations. However, the functions of only 30.5% (40/131) of the mutations have been identified.

**Figure 3:**
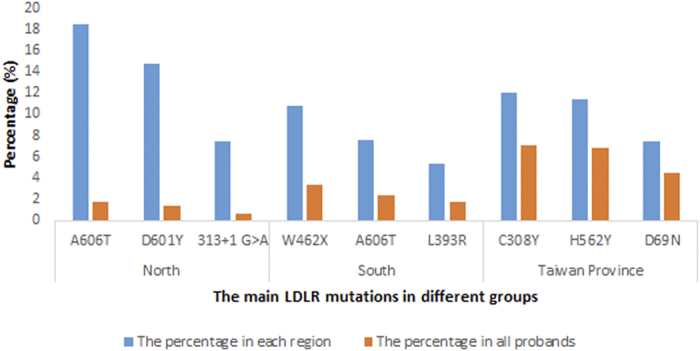
Frequencies of the main LDLR mutations in different regions of China.

Among all of the 131 mutations, three mutations were reported more frequently: C308Y (c.986G > A, p.Cys329Tyr), H562Y (c.1747C > T, His583Tyr) and A606T (c.1879G > A, p.Ala627Thr) (Table 1), which accounted for 23% of the probands. Furthermore, three groups were identified based on their different geographical locations in China to determine whether there were characteristic mutations in each region: the North of China group, the South of China group, and the Taiwan Province group. Because of the special historical background of Taiwan Province, it was classified as an independent group. The three most common mutations in the North of China group were A606T (c.1879G > A, p.Ala627Thr), D601Y (c.1864G > T, p.Asp622Tyr) and 313 + 1G > A, and their frequencies represented 18.5%, 14.8%, and 7.4% of the probands in the North, respectively. The main mutations in the South of China group were not the same as in the North; they were W462X (c.1448G > A, p.Trp483X), A606T (c.1879G > A, p.Ala627Thr), and L393R (c.1241T > G, p.Leu414Arg), accounting for 10.7%, 7.5%, and 5.4%, respectively, of the probands in the South. However, there was a large difference between Taiwan Province and the other groups. The three most common mutations in Taiwan Province were C308Y (c.986G > A, p.Cys329Tyr), H562Y (c.1747C > T, His583Tyr), and D69N (c.268G > A, p.Asp90Asn), which accounted for 12%, 11.4%, and 7.4% of the Taiwanese probands, respectively (Fig. 3).

In addition, we identified 30 mutations that were not recorded in the above two LDLR databases (Table 2). The most common type of mutation was a missense mutation (63.3%); frame-shift mutations accounted for 20% of the mutations. Functional studies have been performed for only 8 mutations. Therefore, the following open access software was used to predict the pathogenicity of the other mutations: (1) PolyPhen 2^16^, (2) SIFT^17^, and (3) Mutation Taster^18^. Because nonsense substitutions and frame-shift rearrangements are known to be pathogenic, we did not perform an *in silico* analysis for these mutations. A total of 13 mutations were analyzed, and most of the mutations were pathogenic, excluding two variants that were identified as non-pathogenic: N494S (c.1544A > G, p. Asn515Ser) and S533L (c.1661C > T, p. Ser554Leu).

### FH in Taiwan Province

Chiou and Charng’s group has been devoted to studying FH in Taiwan Province and has contributed greatly to the study of FH in the Chinese population. Since 2003, Charng has screened 170 unrelated hyperlipidemic Chinese patients using DNA screening and identified 10 mutations and two polymorphisms^19^. To date, a total of 175 probands, including 64 mutations, have been identified (Supplemental Table S1 online). Regarding the regional distribution, these probands could account for the highest percentage of probands in China, representing 59.3% of the total probands (Supplemental Fig. S2 online). There are three primary FH mutations in Taiwan Province. (1) C308Y (c.986G > A, p.Cys329Tyr) (21/175), in which the normal amino acid is a highly conserved cysteine residue at position 308. This variant may cause misfolding of the LDLR protein; the mutated protein localizes in the endoplasmic reticulum (ER) and exerts only 31% LDLR activity when transiently expressed in COS-7 cells^19^. However, this mutation has also been identified in Russia^20^, the Philippines^21^, Hong Kong^22^, and the Netherlands^23^. (2) H562Y (c.1747C > T, His583Tyr) (20/175) was first identified in a Chinese patient with FH by researchers from Jiangsu Province^14^. This mutation is located in the EGF-precursor domain, and as a result, only 50% of the mature protein is successfully synthesized. In addition, LDL binding is also limited when the mutated gene is transfected into CHO cells^14^. A haplotype analysis showed that the common ancestor of this mutation may have originated from an indigenous population (Yueh) living on the southeast coast of China, including Jiangsu, Guangdong and Fujian Provinces^24^. (3) The D69N mutation (c.268G > A, p.Asp90Asn) (13/175) is located in a highly conserved region in the LDLR binding domain. In this mutation, the LDLR protein is retained in the ER and exerts only 55% of its normal activity. This mutation had also been identified in Hong Kong and Malaysia^22^,^25^, and the results of a haplotype analysis suggested that it may have originated in southern China^19^.

### FH in Hong Kong

Researchers first reported FH patients with mutations in Hong Kong in 1998^22^. The authors used single-strand conformation polymorphism (SSCP) and direct DNA sequencing to identify a total of 18 mutations in 30 Chinese patients with potential FH. In that study, three recurrent mutations were identified: L393R (3/21, c.1241T > G, p.Leu414Arg), C308Y (2/21, c.986G > A, p. Cys329Tyr) and V408M (2/21, c.1285G > A, p.Val429Met). The L393R mutation is located in exon 9 and may belong to class 5 because its functional domain is in the EGF spacer. The *in silico* analysis revealed that this mutation is likely to be pathogenic (Table 2). The V408M mutation is also present in exon 9 and was first reported in Afrikaans patients^26^. A functional study in CHO cells showed < 2% LDLR activity in homozygotes. Unfortunately, there are no additional published studies on FH mutations in Hong Kong, and therefore, the above three recurrent mutations could not be implicated in common mutations due to the founder effect.

### FH in mainland China

To date, 99 probands including 76 different mutations in 14 areas of mainland China have been reported (Supplemental Fig. S2 online). Regarding the regional distribution, the probands that originated from Guangdong Province have been reported most frequently, although the related reports are not from China^25^,^27^. Other provinces that reported more than 8 probands included Jiangsu, Beijing, Anhui, Hubei, and Henan Provinces. Among all of the gene mutations, the most common ten mutations in mainland China are shown in Supplemental Figure S3A online. When the data obtained for Taiwan Province and Hong Kong are included in the analysis, the top ten most common mutations change (Supplemental Fig. S3B online). However, we do not discuss every mutation in each city because the numbers of probands in each city were not sufficient to confirm the origin of each mutation. Therefore, we analyzed only the three primary mutations in mainland China (Table 1).

The three primary mutations are A606T (c.1879G > A, p.Ala627Thr), W462X (c.1448G > A, p.Trp483X) and D601Y (c.1864G > T, p.Asp622Tyr), which account for 29.3% of probands in mainland China. (1) The first mutation, A606T, is located in the thirteenth exon encoding the EGF precursor homology domain. This mutation can lead to reductions in the mobility of the mature LDLR protein, precursor protein synthesis, LDL binding, and the rate of recirculation^14^. (2) The second mutation, W462X, includes a G-to-A change at nucleotide 1448 in exon 10 and was first reported in Jiangsu Province in China^14^. Our group discovered that the W462X mutation results in a truncated LDLR protein with only 17% LDL binding activity and 39% LDL internalization, despite a residual LDLR expression level of 81%^7^. (3) The third mutation, D601Y, is also located in the thirteenth exon and was first reported by our group in 2008 in China^28^. Flow cytometric analyses of lymphocytes from HoFH patients revealed that the mutation is associated with LDLR expression of only 13.6%, LDL binding of 21.1% and LDL internalization of 94.3% in comparison to the controls^28^.

### Other mutations in FH in China

In addition to the LDLR mutations, other mutations can also cause FH, such as mutations in the apoB and PCSK9 genes. However, only 12 studies have reported apoB and PCSK9 mutations in China. The most common apoB mutation is R3500W (c.10707 C > T, 50/56), which is located in exon 26 and was first reported by Huang *et al.* in 1998^29^. The other apoB mutations were R3500Q (E26, c.10708 G > A, 3/56), T3540M (E26, c.10828 C > T, 1/56) and R4019W (E29, c.12265 C > T, 2/56). Six mutations in PCSK9 have been reported: intron 2 T > G, R306S, V312S, V312F, R319E, and D320N (Supplemental Table S2 online). Functional experiments have been reported for only R306S, and the results revealed that mature LDL-R was significantly decreased by 12% following transfection of the R306S mutant into Bel-7402 cells^30^.

### The clinical phenotypes of FH in China

The first study to discuss phenotypic variations in HeFH patients in China was published in 1998, in which Chinese patients with FH were compared with patients with the same or similar mutations in Canada^27^. The study reported that the total cholesterol (TC) and LDL-C values in patients living in Canada were higher than those in patients living in China, similar to the incidence rate of coronary artery disease (CAD). These results suggested that environmental factors are very important for the clinical phenotype of FH patients. Later, researchers from Taiwan Province and Hong Kong reported the characteristics of FH patients in their cities^31^,^32^. Their data showed that the TC levels in Taiwan Province (9.1 mmol/L) were similar to those in Hong Kong (9.4 mmol/L), higher than the levels in Japan (8.4 mmol/L)^33^, Malaysia (7.7 mmol/L)^34^, Australia (6.46 mmol/L)^35^ and the Netherlands (5.97 mmol/L)^36^, and lower than the levels in the UK (10.26 mmol/L)^37^ and Spain (10.79 mmol/L)^38^ (Table 3). The data obtained for HeFH patients in mainland China were selected from 1998 and showed that both the TC and LDL-C levels were lower compared with the other countries, excluding the Netherlands^27^ (Table 3). Moreover, the higher xanthomata rates of FH patients in Japan and Malaysian were accompanied by a significant increase in CVD events. However, patients with FH in Australia and the Netherlands exhibited both lower TC and LDL-C values and a lower rate of CVD.

### The relationship between mutations and clinical phenotypes

The data obtained for clinical features and lipid values in the included studies were also collected. A total of 524 patients were diagnosed with FH by gene screening, but clinical features or (and) lipid values were reported for only 355 patients. Table 4 shows the clinical characteristics of the different types of patients with FH. Among these data, we found that both HoFH and compound heterozygous FH patients were younger than the other groups, and they were also more vulnerable to corneal arcus, xanthoma and pCHD. Moreover, the levels of both TC and LDL-C were higher in HoFH than in the compound heterozygous, heterozygous and mutation-negative groups. Interestingly, the heterozygous patients had lower TC and LDL-C levels compared with the mutation-negative group. In addition, 6 patients showed a clinical homozygous phenotype but were genetically heterozygous and presented higher TC and LDL-C values compared with homozygous patients (Table 4). We also evaluated the clinical characteristics associated with different types of gene mutations (Supplemental Table S3 online). Clearly, the TC and LDL-C levels of HoFH patients were higher than those of HeFH patients in each group. Moreover, frameshift mutations appeared to be associated with higher TC levels in either HoFH or HeFH patients compared with the other groups.

### The diagnostic criteria for FH and screening methods in China

There are three internationally recognized clinical diagnostic criteria for FH worldwide: the DLCN criteria, the “Simon Broome” UK-FH register criteria, and the MEDPED System^1^. Scholars in Hong Kong and Taiwan Province have adopted one of the above clinical diagnostic criteria for FH, whereas in mainland China, scholars use different clinical diagnostic criteria based on the book “Clinical coronary heart disease”, which was published in 1998^39^. If patients and/or their relatives have tendon xanthoma and meet one of the following conditions, they may be diagnosed with FH: 1. child <16 years of age with plasma TC levels >6.7 mmol/L (300 mg/dl); 2. adult with plasma TC levels >7.8 mmol/L (260 mg/dl); or 3. patient with plasma LDL-C values >4.9 mmol/L (190 mg/dl). When patients with tendon xanthoma have a TC level >16 mmol/L (60 mg/dl), they are diagnosed as HoFH; otherwise, they may be diagnosed as HeFH.

The gene diagnoses are also important. The Chinese researcher Sun first used SSCP and Southern blotting to detect LDLR mutations in Chinese patients in 1994, but all of the experiments were completed in the UK^14^. Other technologies have also been employed, such as long-chain polymerase chain reaction^40^ and polymerase chain reaction-single strand conformation polymorphism (PCR-SSCP)^41^. Although these methods can detect whether variations are present, they are not capable of confirming the sites of the mutations. In 2001, Wang *et al.* published an SSCP and DNA sequencing analysis to screen all 18 exons and exon-intron boundaries in the LDLR gene. The authors identified one mutation in exon 6 (c.850 T > C, p. Cys284Arg)^42^. Our group later conducted touch-down PCR together with DNA sequencing and denaturing high-performance liquid chromatography (DHPLC) to detect LDLR mutations^6^,^43^. We also conducted the first study to utilize an SNP-based genome-wide linkage scan and whole/targeted exome sequencing technologies to detect LDLR mutations in China^8^,^10^,^44^.

## Discussion

In this systematic review, we analyzed the characteristics, distribution, gene frequency, and relationship between the genotype and phenotype of LDLR gene mutations in the Chinese population. A total of 74 studies, involving 586 patients (including 357 probands) with 141 related mutations in FH, were included in this review. In summary, the findings from these studies indicate that although FH is widely distributed in most regions of China, the diagnostic and therapeutic rates of FH were less than 1%. This finding suggests that most doctors and people lack an understanding of FH and also pay insufficient attention to the occurrence of FH in China.

To date, only one study has estimated the prevalence and treatment of FH, which included 9,324 Chinese subjects from the Jiangsu Nutrition Study. The researchers reported that the prevalence of probable/definite FH was 0.28% (1.4/500) based on the modified DLCN definition, only 15.9% of the patients were receiving drug treatment, and none of the patients achieved the LDL target value (<2.5 mmol/L)^4^. This study also demonstrated that FH patients had more than a 15-fold increased risk of cardiovascular disease compared with patients without FH. The authors suggested that FH is not rare in the Chinese population and remains largely unidentified. Chiou reported that the frequency of apoB gene mutations is less than 10% in Taiwan Province^24^. In addition, the frequency of PCSK9 gene mutations appeared to be less than 5% because Song *et al.* identified only 5 mutations in 100 hyperlipidemia patients and did not detect any LDLR or apoB mutations^45^. However, a large-scale clinical study for FH screening in mainland China should be performed to confirm these results.

It is well known that high-frequency LDLR gene mutations can aid in the screening for FH. For example, the S472RfsX44 mutation (in exon 10) is responsible for approximately 42% of the patients with FH in Tunisia^46^. The nine most common mutations have been shown to be responsible for 66.5% of the patients with FH in the Netherlands^23^. Our review revealed that the five most common mutations (C308Y, H562Y, A606T, D69N, and W462X) could explain 31.9% of the FH probands in China (Supplemental Fig. 3 online). Furthermore, the three most common mutations in mainland China encompass 43.8% of the provinces with reported mutations, and these mutations have only been reported in the Chinese population, excluding the W462X mutation, which is also found in Australia^47^ (Supplemental Fig. 3 online). This finding suggests that the above mutations may be the most specific and common mutations in China, which may be useful for FH screening in the future. However, owing to the small sample size, future large-scale screening trials should be conducted to confirm these conclusions.

In the analysis of clinical phenotypes, we discovered that levels of TC and LDL-C in patients from Hong Kong and Taiwan Province were similar to the levels measured in patients in Western countries and higher than those in patients in Asia and parts of Europe. One possible explanation for this finding is that the diets and lifestyles of these patients are similar to those of patients in Western countries. In analyses of the relationship between mutations and clinical phenotypes, we discovered two interesting phenomena: (1) the mutation-negative group had lipid values that were comparable to the HeFH group and (2) there were 6 probands that were diagnosed as having a HeFH genotype but actually had a clinical homozygous phenotype. These data suggest that there may be other pathogenic genes or many minor genes that participate in the development of disease, as suggested in a previous study^48^. Therefore, future studies should be conducted to confirm these phenomena.

There are several limitations to this review. First, different nomenclatures were utilized for the LDLR mutation in the included studies. Hence, we unified the nomenclature of the mutations in this review. Second, some of the studies did not mention the origin of the probands. Consequently, we used the location of the first author affiliation. Thus, there are some deviations in the regional distribution of the mutations. Third, we reviewed and analyzed the clinical data obtained for the FH patients. However, the conclusions from these data analyses should be treated with caution because this was a systematic review, and all the data were analyzed in a secondary manner. Finally, we included only LDLR mutations identified in China, which may result in regional limitations.

## Conclusions

In recent years, interest in FH has grown among the international community, and many countries have published FH guidelines to facilitate improved management and treatment of patients with FH^1^,^3^. The current review identified only 357 probands in China, including 131 LDLR mutations, 4 apoB mutations, and 6 PCSK9 mutations, which may provide information that is specific to China for inclusion in the international FH database. However, the functions of these mutations and which mutations influence the prognosis of patients remain unknown. Furthermore, whether other genes or SNPs exist that may cause this disease or influence the treatment of FH remains to be discovered. These questions should be addressed by researchers in China in future studies. However, a reliance on doctors and laboratory personnel to address FH is insufficient, and it will be critical to raise public awareness of FH. Finally, China is one of the most populous countries in the world and may have a greater genetic burden resulting in more patients with FH. We hope that the present review will raise awareness among doctors and the public and promote the initiation of future studies to explore the diagnosis and treatment of FH.

## Methods

### Search strategy

Using the computerized literature databases of PubMed, Embase, Wanfang (Chinese), the Chinese National Knowledge Infrastructure (CNKI, Chinese), and the Chinese Biological and Medical database (CBM, Chinese), and public data were limited to December 2014. The Medical Subject Heading terms and the following key words were used: “familial hypercholesterolemia”, “Chinese”, “China”, “Hong Kong”, and “Taiwan”. Only English and Chinese language articles were included in the analysis.

### Selection and definition of gene nomenclature

The inclusion criteria for the included studies were as follows: any reports of pathogenic mutations in FH, such as LDLR, APOB, and PCSK9 gene mutations. Two nomenclatures were used to describe LDLR gene mutations in the LOVD databases. The first nomenclature was based on the guidelines from www.hgvs.org/mutnomen, starting with nucleotide + 1 as the A in the ATG translation initiation codon^49^. The second nomenclature begins with negative numbers from the exon 1 codons^50^. In this review, we adopted the second nomenclature as the primary form and listed the alternate nomenclature in brackets.

### Data extraction

Two independent researchers (L.Y.S. and Y.F.D.) reviewed the relative articles based on the abstracts and full texts to assess their eligibility. One researcher (F.Z.) collected information concerning the LDLR mutations, including the exons, cDNA, proteins, distribution, and function of the LDLR gene mutations. If the articles reported sequencing information, then the information for mutations was assessed between the images and original nucleotide sequence. If the information differed between the images and text describing the mutations, we collected information regarding mutations from the sequencing results. The clinical characteristics included age, sex, and lipid values, among others. All authors of this review were consulted if additional data were collected or the data were unclear.

### Mutation prediction

Novel LDLR variants were assessed by in *silico* mutation prediction tools, including PolyPhen2^16^, SIFT^17^, and Mutation Taster^18^.

### Statistical analysis

All of the statistical analyses were performed using SPSS version 18.0 (SPSS, Inc., Chicago, Illinois). Measurement data are presented as the mean ± SD, and count data are presented as rates.

## Additional Information

**How to cite this article**: Jiang, L. *et al.* The distribution and characteristics of LDL receptor mutations in China: A systematic review. *Sci. Rep.* **5**, 17272; doi: 10.1038/srep17272 (2015).

## Supplementary Material

Supplementary Information

## Figures and Tables

**Table 1:** Characteristics of the primary mutations in China.

Group*	Primary Mutations					Regional distribution in China	Also found in
	Exon	cDNA	Protein	Probands	Function		
Group 1	E7	c.986G > A	p.C308Y (Cys329Tyr)	26	31% LDLR activity in transfected COS cells	Beijing, Guangdong Province, Taiwan Province, Hong Kong	Russia^20^, Philippines^21^, Hong Kong^22^, and the Netherlands^23^
	E12	c.1747C > T	p. H562Y (His583Tyr)	23	Precursor accumulates; approximately 50% mature protein	Jiangsu Province, Shanghai, Taiwan Province	Philippines^21^
	E13	c.1879G > A	p. A606T (Ala627Thr)	19	bind LDL slow processing recycling defective	Anhui Province, Beijing, Henan Province, Hebei Province, Hubei Province, Jiangsu Province, Taiwan Province, Hong Kong	NA
Group 2	E13	c.1879G > A	p. A606T (Ala627Thr)	13	bind LDL slow processing recycling defective	Anhui Province, Beijing, Henan Province, Hebei Province, Hubei Province, Jiangsu Province	NA
	E10	c.1448G > A	p. W462X (Trp483X)	10	17% LDLR binding and 39% internalization activity in transfected 293T cells	Anhui Province, Guangdong Province, Hubei Province, Jiangsu Province, Zhejiang Province	Austria^47^
	E13	c.1864G > T	p. D601Y (Asp622Tyr)	6	13.6% LDLR expression and 21.1% binding activity in peripheral blood lymphocytes from patients	Anhui Province, Beijing, Hubei Province, Shanxi Province	NA

*Group 1 included Hong Kong, Taiwan Province and mainland China; Group 2 only included mainland China.

**Table 2:** Characteristics of mutations that were not recorded in LDLR databases and *in silico* predictions.

Exon	cDNA	Protein	position	PhlyPhen−2 (Hum Div)	PhlyPhen−2 (Hum Var)	SIFT	Mutation Taster	Overall	LDLR Activity*
E1	C.−44C > T								NA
E1	64delG		11089612				Disease causing	Pathogenic	NA
E3	310T > C	C83R	11102783	Probably Damaging	Probably Damaging	Damaging	Disease causing	Pathogenic	NA
E4	c.383G > A	C107Y	11105289	Probably Damaging	Probably Damaging	Damaging	Disease causing	Pathogenic	NA
E4	c.385G > T	D108Y	11105291	Probably Damaging	Probably Damaging	Damaging	Disease causing	Pathogenic	NA
E4	c.444T > A	C127X	11105350				Disease causing	Pathogenic	NA
E4	551_553delGTAinsTT							Pathogenic	NA
E6	c.889delA		11107463				Disease causing	Pathogenic	14% LDLR activity in peripheral blood lymphocytes from HoFH patients
E6	c.890A > C	N276T	11107464	Possibly Damaging	Possibly Damaging	Damaging	Disease causing	Pathogenic	NA
E6	c.892delA		11107466				Disease causing	Pathogenic	NA
E7	c.1054 T > A	C331S	11110765	Probably Damaging	Probably Damaging	Damaging	Disease causing	Pathogenic	NA
E8	c.1100 T > A	L346H	11111553	Possibly Damaging	Possibly Damaging	Damaging	Disease causing	Pathogenic	NA
E8	c.1129 T > G	C356G	11111582					Pathogenic	57% LDLR binding and 52% internalization activity in transfected 293T cells
E9	c.1304A > G	E414G	11113395	Probably Damaging	Probably Damaging	Damaging	Disease causing	Pathogenic	NA
E9	c.1329delG		11113420				Disease causing	Pathogenic	NA
E10	c.1439C > T	A459V	11113615					Pathogenic	39% LDLR expression, 63% binding and 76% internalization activity in peripheral blood lymphocytes from HoFH patients
E10	c.1544A > G	N494S	11113720	Bening	Bening	Tolerated	Polymorphism	Non-pathogenic	NA
Intron10	c.1586 + 1 G > T								NA
Intron10	c.1586 + 5 G > C								NA
E11	c.1592T > A	M510K	11116099					Pathogenic	31.6% LDLR binding and 36.2% internalization activity in transfected COS-7 cells
E11	c.1597T > C	W512R	11116104					Pathogenic	0–6% LDLR activity in transfected COS cells
E11	c.1661C > T	S533L	11116168	Possibly Damaging	Bening	Tolerated	Polymorphism	Non-pathogenic	NA
E12	c.1757C > A	S565X	11116910				Disease causing	Pathogenic	16% LDLR binding and 19% internalization activity in transfected 293T cells
E13	c.1849A > G	K596E	11120092	Bening	Bening	Damaging	Disease causing	Pathogenic	NA
E13	c.1864G > T	D601Y	11120110					Pathogenic	13.6% LDLR expression and 21.1% binding activity in peripheral blood lymphocytes from patients
E13	c.1877A > G	E605G	11120123	Possibly Damaging	Possibly Damaging	Damaging	Disease causing	Pathogenic	NA
E14	c.2021A > G	N653S	11120403	Bening	Bening	Damaging	Disease causing	Pathogenic	NA
E14	c.2075C > G	P671G	1120457	Probably Damaging	Probably Damaging	Damaging	Disease causing	Pathogenic	NA
E17	2400insG		11129563				Disease causing	Pathogenic	NA
E17	c.2443C > T	L794F	11129566					Pathogenic	Normal LDLR binding, but 3% internalization activity in skin fibroblast from homozygous FH

All references are showed in supplemental table S1. *Only be reported in China.

**Table 3:** Clinical features and blood lipid values of FH in different countries.

Countries	TC(mmol/L)	LDL-C (mmol/L)	TG(mmol/L)	HDL-C (mmol/L)	Xanthomata (%)	Corneal arcus(%)	CVD(%)
Taiwan Province^31^	9.1	6.83	1.31	1.45	23.5	NA	24.5
Hong Kong^32^	9.41	7.49	1.3	1.35	50	49.1	13
Japan^33^	8.4	6.4	1.49	1.22	87	38	24
Malaysian^34^	7.7	5.1	1.9	1.3	50.6	50.6	80.5
Australia^35^	6.46	4.42	1.2	1.3	18.6	26.1	9.6
Netherlands^36^	5.97	4.13	1.1	1.2	NA	NA	9.2
UK^37^	10.26	4.69*	1.34*	1.36*	28.6	16.7	19.4
Spain^38^	10.79	8.68	1.2	1.34	28.5	NA	21.9
Mainland China (1998)^27^	6.09	4.35	1.23	1.32	0	NA	0

All lipid value were used as average value in each studies. CVD, cardiovascular disease; HDL-C, high density lipoprotein-cholesterol; LDL-C, low density lipoprotein-cholesterol; TC, total cholesterol; TG, triglyceride.

^*^Treated lipid values.

**Table 4:** Relationship between mutations and clinical phenotypes.

	Homozygous FH	Compound Heterozygous FH	Heterozygous FH	Mutation (−)	Heterozygous but Clinical Homozygous FH*
Numbers	42	19	285	18	6
Ages	18.3 ± 14.9	13 ± 11	39.9 ± 16.3	35.8 ± 13.8	18.8 ± 18.5
Male (%)	45.2%	57.9%	48.4%	66.7%	50%
Corneal arcus (%, n/N)	76.5% (13/17)	57.1% (4/7)	34.8% (23/66)	11.1% (1/9)	0 (1)
Xanthoma (%, n/N)	95% (38/40)	100% (19/19)	31.6% (54/171)	50% (9/18)	100% (5/5)
CVD (%, n/N)	57.1% (20/35)	57.9% (11/19)	22.2% (37/167)	11.1% (2/18)	20% (1/5)
TC (mmol/L, n/N)	17.47 ± 4.2 (42/42)	15.87 ± 4.27 (19/19)	8.21 ± 2.02 (285/285)	9.47 ± 4.06 (18/18)	18.03 ± 4.75 (6/6)
LDL-C (mmol/L, n/N)	15.02 ± 4.63 (38/42)	13.07 ± 3.29 (19/19)	6.17 ± 1.91 (245/285)	7.83 ± 4.12 (18/18)	15.93 ± 3.6 (6/6)
TG (mmol/L, n/N)	1.3 ± 0.67 (34/42)	1.25 ± 0.63 (17/19)	1.53 ± 1.07 (239/285)	1.24 ± 0.45 (18/18)	1.61 ± 0.72 (5/6)
HDL (mmol/L, n/N)	1.14 ± 0.42 (36/42)	1.28 ± 0.63 (19/19)	1.37 ± 0.69 (223/285)	1.16 ± ± 0.32 (18/18)	0.65 ± 0.27 (5/6)

n: the number of patients with recorded information; N: all the patients of each group.

^*^this group included patients which showed clinical homozygous phenotype but were genetically heterozygous.

## References

[b1] Watts G. F. *et al.* Integrated guidance on the care of familial hypercholesterolaemia from the International FH Foundation. Int J Cardiol 171, 309–325 (2014). 24418289 10.1016/j.ijcard.2013.11.025

[b2] Starr B. *et al.* Development of sensitive and specific age- and gender-specific low-density lipoprotein cholesterol cutoffs for diagnosis of first-degree relatives with familial hypercholesterolaemia in cascade testing. Clin Chem Lab Med 46, 791–803 (2008). 18601600 10.1515/CCLM.2008.135

[b3] Nordestgaard B. G. *et al.* Familial hypercholesterolaemia is underdiagnosed and undertreated in the general population: guidance for clinicians to prevent coronary heart disease: consensus statement of the European Atherosclerosis Society. Eur Heart J 34, 3478–3490a (2013). 23956253 10.1093/eurheartj/eht273 PMC3844152

[b4] Shi Z. *et al.* Familial hypercholesterolemia in China: prevalence and evidence of underdetection and undertreatment in a community population. Int J Cardiol 174, 834–836 (2014). 24801084 10.1016/j.ijcard.2014.04.165

[b5] Dai Y. F., Sun L. Y., Zhang X. B. & Wang L. Y. [Research progression of LDLR mutations in Chinese Familial hypercholesterolemia]. Yi Chuan 33, 1–8 (2011). 21377952 10.3724/sp.j.1005.2011.00001

[b6] Cao S. *et al.* Analysis of low-density lipoprotein receptor gene mutations in a Chinese patient with clinically homozygous familial hypercholesterolemia. Chin Med J (Engl) 116, 1535–1538 (2003). 14570618

[b7] Wang L. *et al.* Mutations in the LDL receptor gene in four Chinese homozygous familial hypercholesterolemia phenotype patients. Nutr Metab Cardiovasc Dis 19, 391–400 (2009). 19073363 10.1016/j.numecd.2008.07.011

[b8] Wang X. *et al.* Genome-wide linkage scan of a pedigree with familial hypercholesterolemia suggests susceptibility loci on chromosomes 3q25-26 and 21q22. PLoS One 6, e24838 (2011). 22022364 10.1371/journal.pone.0024838 PMC3194805

[b9] Wang H. *et al.* Functional characterization of two low-density lipoprotein receptor gene mutations in two Chinese patients with familial hypercholesterolemia. PLoS One 9, e92703 (2014). 24671153 10.1371/journal.pone.0092703 PMC3966815

[b10] Wu W. F., Sun L. Y., Pan X. D., Yang S. W. & Wang L. Y. Use of targeted exome sequencing in genetic diagnosis of chinese familial hypercholesterolemia. PLoS ONE 9, (2014). 10.1371/journal.pone.0094697 PMC3983231 24722143

[b11] Ping C. S. & Foon C. L. Essential familial hypercholesterolemia in a Chinese family. The Journal of the Singapore Paediatric Society 13, 101–107 (1971). 5149805

[b12] Cai H. J., Fan L. M. & Huang M. G. Homozygous familial hypercholesterolemic patients in China. Atherosclerosis 57, 303–312 (1985). 4084360 10.1016/0021-9150(85)90042-5

[b13] Hobbs H. H., Brown M. S. & Goldstein J. L. Molecular genetics of the LDL receptor gene in familial hypercholesterolemia. Hum Mutat 1, 445–466 (1992). 1301956 10.1002/humu.1380010602

[b14] Sun X. M. *et al.* Familial hypercholesterolemia in China: Identification of mutations in the LDL-receptor gene that result in a receptor-negative phenotype. Arteriosclerosis and Thrombosis 14, 85–94 (1994). 7903864 10.1161/01.atv.14.1.85

[b15] Usifo E. *et al.* Low-density lipoprotein receptor gene familial hypercholesterolemia variant database: update and pathological assessment. Ann Hum Genet 76, 387–401 (2012). 22881376 10.1111/j.1469-1809.2012.00724.x

[b16] Adzhubei I. A. *et al.* A method and server for predicting damaging missense mutations. Nat Methods 7, 248–249 (2010). 20354512 10.1038/nmeth0410-248 PMC2855889

[b17] Leigh S. E., Foster A. H., Whittall R. A., Hubbart C. S. & Humphries S. E. Update and analysis of the University College London low density lipoprotein receptor familial hypercholesterolemia database. Ann Hum Genet 72, 485–498 (2008). 18325082 10.1111/j.1469-1809.2008.00436.x

[b18] Schwarz J. M., Rodelsperger C., Schuelke M. & Seelow D. MutationTaster evaluates disease-causing potential of sequence alterations. Nat Methods 7, 575–576 (2010). 20676075 10.1038/nmeth0810-575

[b19] Chang J. H. *et al.* Identification and characterization of LDL receptor gene mutations in hyperlipidemic Chinese. J Lipid Res 44, 1850–1858 (2003). 12837857 10.1194/jlr.M200470-JLR200

[b20] Zakharova F. M. *et al.* Familial hypercholesterolemia in St-Petersburg: the known and novel mutations found in the low density lipoprotein receptor gene in Russia. BMC Med Genet 6, 6 (2005). 15701167 10.1186/1471-2350-6-6 PMC551615

[b21] Punzalan F. E. *et al.* Low density lipoprotein–receptor (LDL-R) gene mutations among Filipinos with familial hypercholesterolemia. J Atheroscler Thromb 12, 276–283 (2005). 16205024 10.5551/jat.12.276

[b22] Mak Y. T. *et al.* Mutations in the low-density lipoprotein receptor gene in Chinese familial hypercholesterolemia patients. Arterioscler Thromb Vasc Biol 18, 1600–1605 (1998). 9763532 10.1161/01.atv.18.10.1600

[b23] Fouchier S. W., Defesche J. C., Umans-Eckenhausen M. W. & Kastelein J. P. The molecular basis of familial hypercholesterolemia in The Netherlands. Hum Genet 109, 602–615 (2001). 11810272 10.1007/s00439-001-0628-8

[b24] Chiou K. R. & Charng M. J. Common mutations of familial hypercholesterolemia patients in Taiwan: characteristics and implications of migrations from southeast China. Gene 498, 100–106 (2012). 22353362 10.1016/j.gene.2012.01.092

[b25] Khoo K. L. *et al.* Low-density lipoprotein receptor gene mutations in a Southeast Asian population with familial hypercholesterolemia. Clin Genet 58, 98–105 (2000). 11005141 10.1034/j.1399-0004.2000.580202.x

[b26] Leitersdorf E., Van der Westhuyzen D. R., Coetzee G. A. & Hobbs H. H. Two common low density lipoprotein receptor gene mutations cause familial hypercholesterolemia in Afrikaners. J Clin Invest 84, 954–961 (1989). 2569482 10.1172/JCI114258 PMC329741

[b27] Pimstone S. N. *et al.* Phenotypic variation in heterozygous familial hypercholesterolemia: a comparison of Chinese patients with the same or similar mutations in the LDL receptor gene in China or Canada. Arterioscler Thromb Vasc Biol 18, 309–315 (1998). 9484998 10.1161/01.atv.18.2.309

[b28] Xu Y. J. *et al.* Analysis of LDL receptor function and gene mutations in familial homozgous hypercholesterolemia (Chinese). Lin Chuang Xin Xue Guan Bing Za Zhi 25, 350–354 (2008).

[b29] Huang J. J., Zhou X., Chen F. & Ha D. W. Screening of Familial Defective Apolipoprotein B-100 (Chinese). Zhong Guo Xun Huan Za Zhi, 30–32 (1998).

[b30] Lin J. *et al.* A novel mutation in proprotein convertase subtilisin/kexin type 9 gene leads to familial hypercholesterolemia in a Chinese family. Chin Med J (Engl) 123, 1133–1138 (2010). 20529551

[b31] Chiou K. R. & Charng M. J. Detection of mutations and large rearrangements of the low-density lipoprotein receptor gene in Taiwanese patients with familial hypercholesterolemia. Am J Cardiol 105, 1752–1758 (2010). 20538126 10.1016/j.amjcard.2010.01.356

[b32] Hu M. *et al.* Heterozygous familial hypercholesterolemia in Hong Kong Chinese. Study of 252 cases. International Journal of Cardiology 167, 762–767 (2013). 22464486 10.1016/j.ijcard.2012.03.048

[b33] Bujo H. *et al.* Clinical features of familial hypercholesterolemia in Japan in a database from 1996-1998 by the research committee of the ministry of health, labour and welfare of Japan. J Atheroscler Thromb 11, 146–151 (2004). 15256765 10.5551/jat.11.146

[b34] Al-Khateeb A., Al-Talib H., Mohamed M. S., Yusof Z. & Zilfalil B. A. Phenotype-genotype analyses of clinically diagnosed Malaysian familial hypercholestrolemic patients. Adv Clin Exp Med 22, 57–67 (2013). 23468263

[b35] Bell D. A. *et al.* Effectiveness of genetic cascade screening for familial hypercholesterolaemia using a centrally co-ordinated clinical service: An Australian experience. Atherosclerosis 239, 93–100 (2015). 25585028 10.1016/j.atherosclerosis.2014.12.036

[b36] Besseling J. *et al.* Severe heterozygous familial hypercholesterolemia and risk for cardiovascular disease: a study of a cohort of 14,000 mutation carriers. Atherosclerosis 233, 219–223 (2014). 24529147 10.1016/j.atherosclerosis.2013.12.020

[b37] Humphries S. E. *et al.* Mutational analysis in UK patients with a clinical diagnosis of familial hypercholesterolaemia: relationship with plasma lipid traits, heart disease risk and utility in relative tracing. J Mol Med (Berl) 84, 203–214 (2006). 16389549 10.1007/s00109-005-0019-z

[b38] Alonso R. *et al.* Cardiovascular disease in familial hypercholesterolaemia: influence of low-density lipoprotein receptor mutation type and classic risk factors. Atherosclerosis 200, 315–321 (2008). 18243212 10.1016/j.atherosclerosis.2007.12.024

[b39] J. C. Z., Q. X. Y. & H.Y. K. Clinical coronary heart disease. Beijing: People’s Military Medical Press, 77 (1998).

[b40] Zhou T. H., Bahring S. & Schuster H. Use long chain polymerase chain reaction to research restriction fragment length polymorphism of low density lipoprotein receptor gene (Chinese) Zhong Hua Yi Xue Za Zhi 76, 465–467 (1996).

[b41] Zhu D. M. *et al.* The application of polymerase chain reaction-single strain conf ormat ion polymorphism in the pedigree analysis of familial hypercholerolemia patients. Zhong Hua Yi Xue Yi Chuan Xue Za Zhi 17, 108–111 (2000). 10751533

[b42] Wang D. *et al.* A Chinese homozygote of familial hypercholesterolemia: identification of a novel C263R mutation in the LDL receptor gene. J Hum Genet 46, 152–154 (2001). 11310584 10.1007/s100380170104

[b43] Wang L. Y. *et al.* Analysis of temperature-modulated high pe rformance liquid chromatography for detecting mutations of LDLR gene in a familial hypercholesterolemia pedigree (Chineses). Zhong Hua Jian Yan Yi Xue Za Zhi 28, 359–363 (2005).

[b44] Sun L. Y. *et al.* Identification of the gene defect responsible for severe hypercholesterolaemia using whole-exome sequencing. Sci Rep 5, 11380 (2015). 26077743 10.1038/srep11380 PMC4468422

[b45] Song G. Y. *et al.* Research of Gene Mutation of Proprotein Convertase Subtilisin /Kexin 9 in Hypercholesterolemia. Zhong Guo Dong Mai Ying Hua Za Zhi, 731–735 (2012).

[b46] Jelassi A. *et al.* Limited mutational heterogeneity in the LDLR gene in familial hypercholesterolemia in Tunisia. Atherosclerosis 203, 449–453 (2009). 18757057 10.1016/j.atherosclerosis.2008.07.011

[b47] Schmidt H. & Kostner G. M. Familial hypercholesterolemia in Austria reflects the multi-ethnic origin of our country. Atherosclerosis 148, 431–432 (2000). 10657581 10.1016/s0021-9150(99)00469-4

[b48] Talmud P. J. *et al.* Use of low-density lipoprotein cholesterol gene score to distinguish patients with polygenic and monogenic familial hypercholesterolaemia: a case-control study. Lancet 381, 1293–1301 (2013). 23433573 10.1016/S0140-6736(12)62127-8

[b49] Villeger L. *et al.* The UMD-LDLR database: additions to the software and 490 new entries to the database. Hum Mutat 20, 81–87 (2002). 12124988 10.1002/humu.10102

[b50] Yamamoto T. *et al.* The human LDL receptor: a cysteine-rich protein with multiple Alu sequences in its mRNA. Cell 39, 27–38 (1984). 6091915 10.1016/0092-8674(84)90188-0

