# Supplementary material for: The distribution and characteristics of LDL receptor mutations in China: A systematic review
Source: Sci Rep. 2015 Nov 26;5:17272. doi: 10.1038/srep17272 (PMC4660303; doi:10.1038/srep17272)
Supplement: Supplementary Information [file srep17272-s1.doc]

### The distribution and characteristics of LDL receptor mutations in China: [A systematic review](http://www.sciencedirect.com/science/article/pii/S0950584914001505)

Long Jiang^1^, Li-Yuan Sun^1,2^, Yan-Fang Dai^1^, Shi-Wei Yang^1^, Feng Zhang^3,4^,

Lu-Ya Wang^1*^

**Supplements**

**Supplemental Table S1 The characteristics of 131 LDLR mutations in China**

**Supplemental Table S2 The characteristics of apoB and PCSK9 mutations in China**

**Supplemental table S3 The clinical characteristics of different mutation types**

**Supplemental Figure S1 The published FH literature for various time quanta in China**

**Supplemental Figure S2 The regional distribution of probands in China**

**Supplemental Figure S3 The number of probands of main mutations (top 10)**

**Supplemental Table S1 The characteristics of 131 LDLR mutations in China**

| **Exon** | **cDNA** | **Protein** | **Distribution** | **LDLR Activity*** | **Remarks** | **References^&^** |
| --- | --- | --- | --- | --- | --- | --- |
| E1 | c.-152C>T |  | Guangdong Province | NA |  | [1] |
| E1 | C.-44C>T |  | Hong Kong | NA |  | [2]new |
| E1 | c.12G>A | W-18X | Taiwan Province, Jiangsu Province | <2% LDLR activity |  | [3-5] |
| E1 | 64delG |  | Taiwan Province | NA |  | [6]new |
| E2 | c.77_78delGA |  | Guangdong Province | NA | 2bp deletion | [1] |
| E2 | c.97C>T | Q12X | Henan Province | NA |  | [7-9] |
| E2 | c.101 G>C | C13S | Taiwan Province | NA |  | [6,10] |
| E3 | 191-?_817+?del |  | Taiwan Province | No LDLR activity in transfected COS cells | 6.6kb del of exons 3-5 | [3] |
| E3 | c.268G>A | D69N | Guangdong Province, Hong Kong, Taiwan Province | 55% LDLR activity in transfected COS cells |  | [1-3,6,10-12] |
| E3 | 310T>C | C83R | Taiwan Province | NA |  | [6]new |
| Intron3 | 313+1G>C |  | Taiwan Province | NA |  | [6] |
| Intron3 | 313+1G>A |  | Guangdong Province, Henan Province | NA |  | [1,13-15] |
| E4 | c.344G>A | R94H | Guangdong Province, Taiwan Province | 64% LDLR activity in transfected COS cells |  | [1,3,6,10] |
| E4 | c.364A>T | I101F | Hong Kong | NA |  | [2] |
| E4 | c.383G>A | C107Y | Taiwan Province | NA |  | [12]new |
| E4 | c.385G>T | D108Y | Beijing | NA |  | [16]new |
| E4 | c.386A>G | D108G | Beijing | NA |  | [17] |
| E4 | c.428G>A | C122Y | Beijing, Anhui Province, Taiwan Province | 59% LDLR binding and 73% internalization activity in transfected 293T cells |  | [6,13,18-20] |
| E4 | c.444T>A | C127X | Beijing | NA |  | [21]new |
| E4 | c.495G>A | W144X | Hunan Province | NA |  | [22] |
| E4 | c.510delC |  | Taiwan Province | NA |  | [6,10] |
| E4 | c.513delC |  | Shanghai | NA |  | [23] |
| E4 | c.516C>G | D151E | Taiwan Province | NA |  | [6,10] |
| E4 | c.517T>C | C152R | Shandong Province | NA |  | [24] |
| E4 | c.550T>C | C163R | Guangdong Province | NA |  | [25] |
| E4 | 551_553delGTAinsTT |  | Shanghai | NA |  | [26]new |
| E4 | 562delT |  | Taiwan Province | NA |  | [6,10] |
| E4 | c.571C>T | G170X | Hong Kong, Taiwan Province | NA |  | [2,12] |
| E4 | c.590G>A | C176Y | Taiwan Province | <2% LDLR activity when heterozygous with FH El Sa |  | [6,10] |
| E4 | c.599T>G | F179C | Taiwan Province | NA |  | [6,10] |
| E4 | c.632_634del |  | Jiangsu Province | Less stable mature protein of faster mobility; does not bind LDL | 3bp deletion (ACT) | [4] |
| E4 | c.656_661del |  | Taiwan Province | NA | 6bp deletion (GCCCCG) | [6,10] |
| E4 | c.664T>C | C201R | Shanghai | NA |  | [27] |
| E4 | c.665G>T | C201F | Hubei Province | 33.2% LDLR expression and 33.5% internalization activity in transfected 293T cells |  | [18,28-29] |
| E4 | c.675delA | K204NfsX40 | Beijing | NA |  | [17] |
| E4 | c.681C>G | D206E | Taiwan Province | NA |  | [6,11] |
| E4 | c.682G>A | E207K | Guangdong Province, Hong Kong, Taiwan Province | 20% LDLR activity in transfected COS cells |  | [2-3,6,25] |
| E4 | c.682G>T | E207X | Jiangsu Province, Taiwan Province | 0-6% LDLR activity in transfected COS cells |  | [4,6,11] |
| E4 | c.683A>C | E207A | Zhejiang Province | 8% LDLR binding activity in HoFH Epstein-Barr transformed cell lines |  | [30-31] |
| E4 | c.691A>C | C210R | Hubei Province | 56% LDLR binding and 62% internalization activity in transfected 293T cells |  | [13] |
| E5 | c.757C>T | R232W | Guangdong Province | NA |  | [1] |
| E5 | c.769C>T | R236W | Taiwan Province | 80% LDLR activity in transfected COS cells |  | [6,10-11] |
| E5 | c.809G>A | C249Y | Jiangsu Province | Slow processing and binding-defective |  | [4] |
| Intron5 | 818－1G>A |  | Hunan Province | NA |  | [22] |
| E6 | c.826T>G | C255G | Jiangsu Province | NA |  | [32] |
| E6 | c.828C>A | C255X | Shanghai, Taiwan Province | NA |  | [6,10,33] |
| E6 | c.850T>C | C263R | Beijing, Taiwan Province | NA |  | [6,34] |
| E6 | c.889delA | N276TfsX73 | Jiangsu Province | 14% LDLR activity in peripheral blood lymphocytes from HoFH patients |  | [35]new |
| E6 | c.890A>C | N276T | Henan Province | NA |  | [7,9]new |
| E6 | c.892delA |  | Henan Province | NA |  | [7,9]new |
| E6 | c.818-?_1186+?del |  | Taiwan Province | 12% LDLR activity in transfected COS cells | 5.3kb del of exons 6-8. | [3,36] |
| E7 | c.947A>G | N295S | Taiwan Province | NA |  | [6,10] |
| E7 | c.986G>A | C308Y | Beijing, Guangdong Province, Hong Kong, Taiwan Province | 31% LDLR activity in transfected COS cells |  | [1-3,6,10-11,37] |
| E7 | c.1016 T>C | P318L | Taiwan Province | NA |  | [6,10] |
| E7 | c.1048 C>T | R329X | Taiwan Province | NA |  | [6,10] |
| E7 | c.1054 T>A | C331S | Taiwan Province | NA |  | [6]new |
| E8 | c.1075 C>T | Q338X | Anhui Province | NA |  | [38] |
| E8 | c.1100 T>A | L346H | Jiangsu Province | NA |  | [32]new |
| E8 | c.1129 T>G | C356G | Jiangsu Province | 57% LDLR binding and 52% internalization activity in transfected 293T cells |  | [19,39]new |
| E8 | c.1132 C>T | Q357X | Guangdong Province | NA |  | [1] |
| E8 | c.1174ins T |  | Taiwan Province | NA |  | [6,10] |
| Intron9 | c.1187-10G>A |  | Tianjin | NA |  | [40] |
| E9 | c.1211C>T | T383I | Beijing, Taiwan Province | 72% LDLR binding and 71% internalization activity in transfected 293T cells |  | [6,13,20] |
| E9 | c.1216C>T | R385W | Taiwan Province | NA |  | [6,10,12] |
| E9 | c.1241T>G | L393R | Guangdong Province, Hong Kong | NA |  | [1-2] |
| E9 | c.1246C>T | R395W | Taiwan Province | NA |  | [6,10-11] |
| E9 | c.1247 G>T | R395L | Taiwan Province | NA |  | [6,10] |
| E9 | c.1257C>A | Y398X | Hubei Province | 5.5% LDLR expression and 20% internalization activity in transfected 293T cells |  | [28,41] |
| E9 | c.1268T>C | I402T | Guangdong Province, Jiangsu Province, Taiwan Province | 59% LDLR binding and 54% internalization activity in transfected COS-7 cells |  | [1,3,19,39] |
| E9 | c.1277T>C | L405P | Hong Kong | NA |  | [2] |
| E9 | c.1285G>A | V408M | Hong Kong, Taiwan Province | NA |  | [2,12] |
| E9 | c.1291G>A | A410T | Taiwan Province | 20% LDLR activity in transfected COS cells |  | [3,6,10] |
| E9 | c.1304A>G | E414G | Jiangsu Province | NA |  | [32]new |
| E9 | c.1322T>C | I420T | Taiwan Province | NA |  | [6,10-11,36] |
| E9 | c.1329G>A | W422X | Taiwan Province | NA |  | [6,10] |
| E9 | c.1329delG |  | Jiangsu Province | NA |  | [32]new |
| Intron9 | c.1358+32C>T |  | Hubei Province | NA |  | [19,28] |
| E10 | c.1384 G>A | V441I | Taiwan Province | NA |  | [6,10] |
| E10 | c.1413G>A | R450R | Shandong Province | NA |  | [42] |
| E10 | c.1432G>A | G457R | Guangdong Province, Hong Kong, Taiwan Province | NA |  | [1-2,6,10-11] |
| E10 | c.1439C>T | A459V | Guangxi | 39% LDLR expression, 63% binding and 76% internalization activity in peripheral blood lymphocytes from HoFH patients |  | [43]new |
| E10 | c.1448G>A | W462X | Anhui Province, Guangdong Province, Hubei Province, Jiangsu Province, Zhejiang Province, Taiwan Province | 17% LDLR binding and 39% internalization activity in transfected 293T cells |  | [4,12,13,25,31,44-51] |
| E10 | c.1474 G>A | D471N | Guangdong Province, Hong Kong, Taiwan Province | NA |  | [1,2,6,10,52] |
| E10 | c.1544 A>G | N494S | Jiangsu Province | NA |  | [32]new |
| Intron10 | c.1586+1 G>T |  | Taiwan Province | NA |  | [6]new |
| Intron10 | c.1586+5 G>C |  | Taiwan Province | NA |  | [12]new |
| E11 | c.1587-?_2140+?del |  | Jiangsu Province | NA | 8kb out of frame deletion of exons 11-14 | [4] |
| E11 | c.1592T>A | M510K | Taiwan Province, Zhejiang Province | 31.6% LDLR binding and 36.2% internalization activity in transfected COS-7 cells |  | [11,31]new |
| E11 | c.1597T>C | W512R | Taiwan Province | 0-6% LDLR activity in transfected COS cells |  | [6,11]new |
| E11 | c.1618G>A | A519T | Taiwan Province | NA |  | [6] |
| E11 | c.1661C>T | S533L | Taiwan Province | NA |  | [6]new |
| E11 | c.1664T>C | L534P | Jiangsu Province | Precursor accumulates; no mature protein |  | [4] |
| E11 | c.1691 A>G | N543S | Taiwan Province | NA |  | [6,10] |
| Intron11 | 1706-1G>T |  | Hong Kong | NA |  | [2] |
| E12 | c.1744C>T | L561F | Anhui Province | NA |  | [19] |
| E12 | c.1747C>T | H562Y | Jiangsu Province, Shanghai, Taiwan Province | Precursor accumulates; approximately 50% mature protein |  | [4,6,10-11,26,36,53] |
| E12 | c.1757C>A | S565X | Shandong Province | 16% LDLR binding and 19% internalization activity in transfected 293T cells |  | [24]new |
| E12 | c.1765G>A | D568N | Taiwan Province | 80% LDLR activity in transfected COS cells |  | [6,10-11] |
| E12 | c.1779delC |  | Hong Kong | NA |  | [2] |
| E12 | c.1783 C>T | R574W | Taiwan Province | NA |  | [6,10] |
| E12 | c.1807 A>T | K582X | Taiwan Province | NA |  | [6,10] |
| E13 | c.1849 A>G | K596E | Jiangsu Province | NA |  | [32]new |
| E13 | c.1851_1862delAGTATTTTGGAC |  | Taiwan Province | NA | 12bp deletion (AGTATTTTGGAC) | [6,10] |
| E13 | c.1864G>A | D601N | Henan Province | NA |  | [18,54] |
| E13 | c.1864G>T | D601Y | Beijing, Hubei Province, Shanxi Province, Anhui Province | 13.6% LDLR expression and 21.1% binding activity in peripheral blood lymphocytes from patients |  | [49,55-58]new |
| E13 | c.1867A>G | I602V | Taiwan Province | normal LDLR activity |  | [3] |
| E13 | c.1877A>G | E605G | Jiangsu Province | NA |  | [32]new |
| E13 | c.1879G>A | A606T | Beijing, Anhui Province, Henan Province, Hong Kong, Hubei Province, Hebei Province, Jiangsu Province, Taiwan Province | bind LDL slow processing recycling defective |  | [2,4,6,10-11, 28,36,46,49,51,59-61] |
| E13 | 1880 C>T | A606V | Hong Kong | NA |  | [2] |
| E13 | 1897 C>T | R612C | Taiwan Province | NA |  | [6,10] |
| E13 | 1907 G>T | G615V | Beijing | 73.6% LDLR expression and 82.6% internalization activity in transfected 293T cells |  | [29] |
| E13 | 1953_1954del |  | Taiwan Province | 0-6% LDLR activity in transfected COS cells | 2bp deletion(TA) | [6,10-11] |
| E14 | 1954_1955del |  | Taiwan Province | NA | 2bp deletion(AT) | [12] |
| E14 | 2012del C |  | Jiangsu Province | <2% LDLR activity |  | [5] |
| E14 | 2015delT |  | Jiangsu Province | Truncated protein accumulates; apparent molecular weight 78 kD |  | [4] |
| E14 | 2021A>G | N653S | Jiangsu Province | NA |  | [32]new |
| E14 | 2030 G>T | C656F | Hong Kong | NA |  | [2] |
| E14 | 2043 C>G | C660W | Taiwan Province | NA |  | [6,36] |
| E14 | 2050G>A | A663T | Guangdong Province | NA |  | [1] |
| E14 | 2054C>T | P664L | Henan Province, Hong Kong, Shanghai, Taiwan Province | NA |  | [2,26,36,62] |
| E14 | 2075C>G | P671G | Beijing | NA |  | [37]new |
| E14 | 2087G>A | C675Y | Guangdong Province | NA |  | [1] |
| E14 | 2099A>G | D679G | Taiwan Province | NA |  | [6] |
| E14 | 2108_2114insTGCTGGC |  | Guangdong Province | NA | 7bp duplication (TGCTGGC) | [1] |
| E15 | 2150C>G | A696G | Taiwan Province | 93% LDLR activity in transfected COS cells |  | [3] |
| E15 | 2215C>T | Q718X | Taiwan Province | NA |  | [6,10] |
| E16 | 2389G>A | V776M | Hong Kong, Shanghai, Taiwan Province | NA |  | [2,6,10,23] |
| E17 | 2400insG |  | Hubei Province | NA |  | [32]new |
| E17 | 2443C>T | L794F | Shandong Province | Normal LDLR binding, but 3% internalization activity in skin fibroblast from homozygous FH |  | [63]new |
| E17 | 2446A>T | K795X | Taiwan Province | NA |  | [6] |
| E17 | 2478delC |  | Jiangsu Province | No protein detected in transfected COS cells |  | [4] |

* only included function of studies in China. ^&^new: not recorded in the two LDLR databases ([www.ucl.ac.uk/ldlr/LOVDv.1.1.0/](http://www.ucl.ac.uk/ldlr/LOVDv.1.1.0/) and <https://grenada.lumc.nl/LOVD2/UCLHeart/home.php?select_db=LDLR>)

**Supplemental Table S2**

**The characteristics of apoB and PCSK9 mutations in China**

| **Gene** | **Exon** | **cDNA** | **Protein** | **Reference** |
| --- | --- | --- | --- | --- |
| ApoB |  |  |  |  |
|  | Exon 26 | c. 10707 C>T | R3500W | [6,64-70] |
|  | Exon 26 | c. 10708 G>A | R3500Q | [68, 71-72] |
|  | Exon 26 | c. 10828 C>T | T3540M | [65] |
|  | Exon 29 | c. 12265 C>T | R4019W | [71] |
| PCSK9 |  |  |  |  |
|  | Intron 2 | T>G |  | [73] |
|  | Exon 6 | c. 916 T>C | R306S | [74] |
|  | Exon 6 | c. 934del G | V312S | [73] |
|  | Exon 6 | c. 934 G>T | V312F | [73] |
|  | Exon 6 | c. 957 G>A | R319E | [73] |
|  | Exon 6 | c. 958 G>A | D320N | [73] |

**Supplemental table S3**

**The clinical characteristics of different mutation types**

|  | Missense Mutations | | Nonsense Mutations | | Frameshift Mutations | |
| --- | --- | --- | --- | --- | --- | --- |
|  | HoFH | HeFH | HoFH | HeFH | HoFH | HeFH |
| Numbers | 28 | 164 | 6 | 48 | 5 | 67 |
| Years | 15.8±13.1 | 41.2±15.6 | 21.2±21.3 | 35.6±15 | 32±17.8 | 37.7±19.4 |
| Male (%) | 50% | 59.4% | 33.3% | 58.3% | 20% | 43.3% |
| Corneal arcus (%, n/N) | 69.2% (9/13) | 40% (20/50) | 100% (2/2) | 28.6% (2/7) | 0 (0/0) | 0 (0/6) |
| Xanthoma (%, n/N) | 96.3% (26/27) | 32.3% (32/99) | 100% (6/6) | 37.5% (12/32) | 100% (3/3) | 36.4% (8/22) |
| CVD (%, n/N) | 47.8% (11/23) | 25.3% (25/99) | 50% (3/6) | 21.2% (7/33) | 100% (3/3) | 9.13 (2/22) |
| TC (mmol/L, n/N) | 16.95±3.67(28/28) | 8.19±2.08(164/164) | 16.86±3.15(6/6) | 8.04±2.79(48/48) | 17.26±2.59 (5/5) | 9.27±3.14(67/67) |
| LDL-C (mmol/L, n/N) | 14.4±3.48(26/28) | 6.24±2.03(140/164) | 14.69±4.09(6/6) | 5.77±2.4(46/48) | 14.55±4.41 (3/5) | 7.43±3.23(53/67) |
| TG (mmol/L, n/N) | 1.29±0.65(22/28) | 1.56±1.01(136/164) | 1.53±1.01(6/6) | 1.7±1.56(45/48) | 1.16±0.26 (4/5) | 1.34±0.63(53/67) |
| HDL (mmol/L, n/N) | 1.19±0.4(25/28) | 1.39±0.87(123/164) | 0.99±0.25(6/6) | 1.18±0.37(43/48) | 1.27±0.9 (3/5) | 1.38±0.39(52/67) |

The compound heterozygous patients were not included. n: the number of patients with recorded information; N: all the patients of each group.


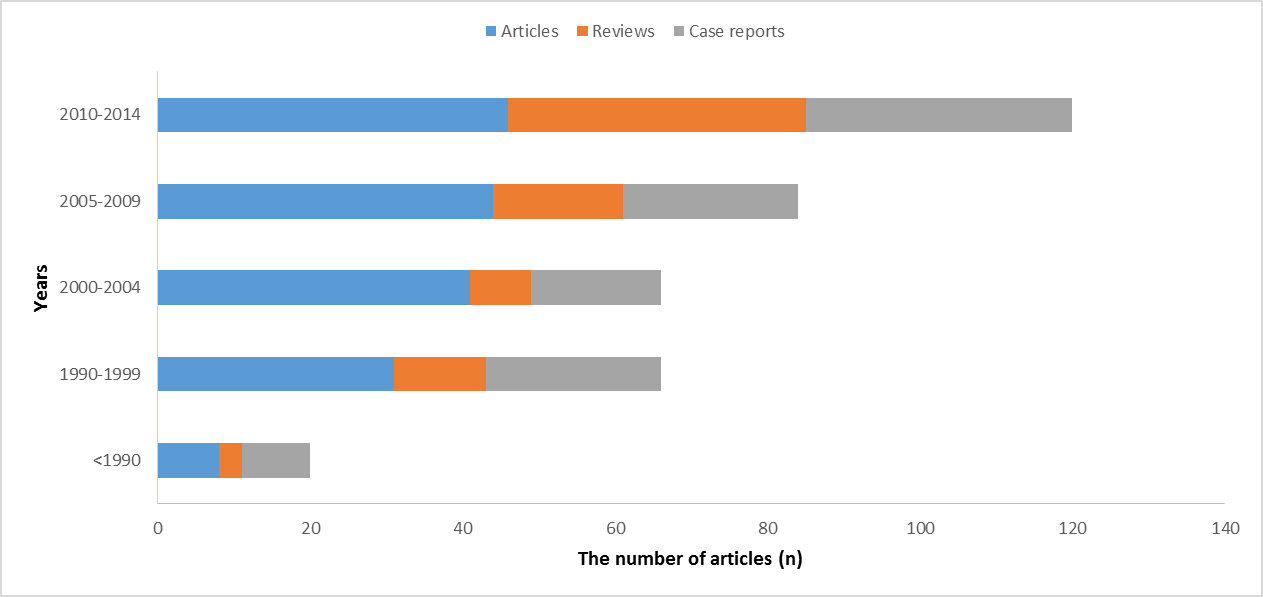


**Supplemental Figure S1**

**The published FH literature for various time quanta in China**


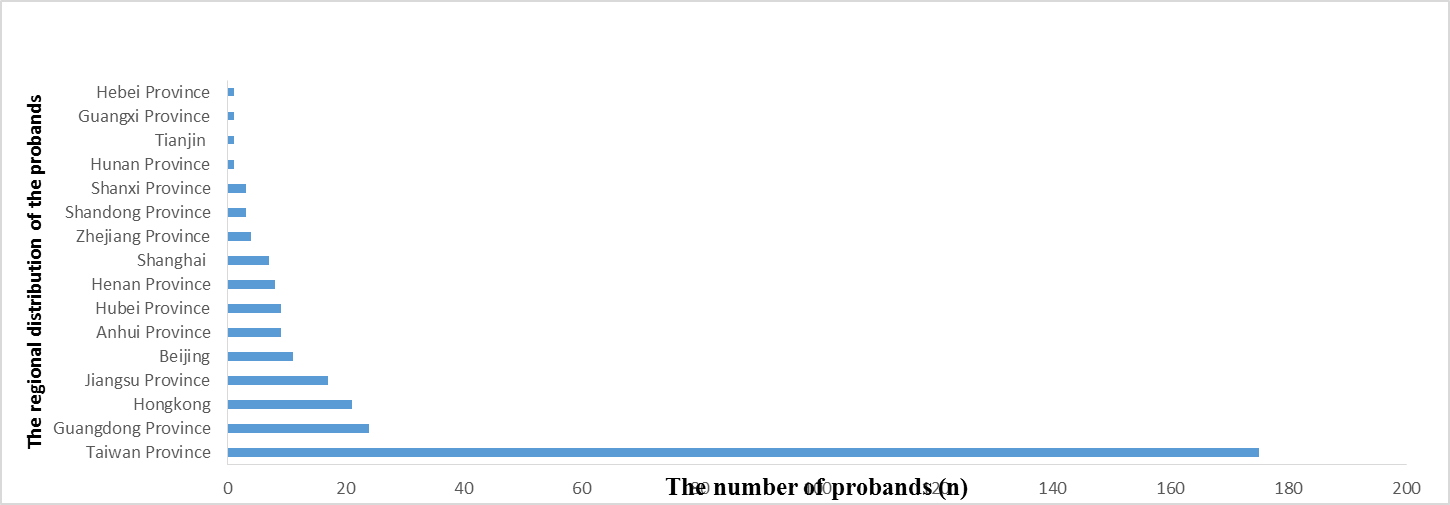


**Supplemental Figure S2**

**The regional distribution of probands in China**


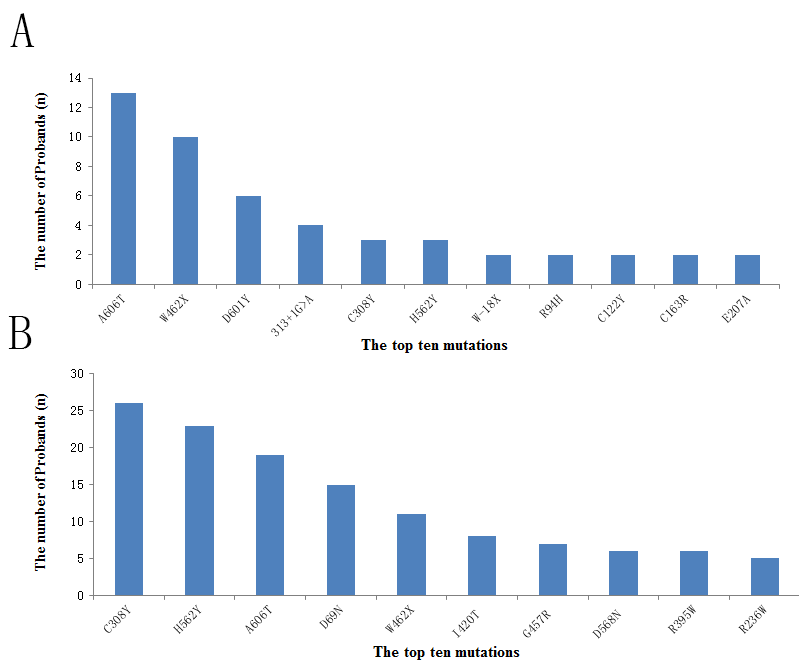


**Supplemental Figure S3**

**The number of probands of main mutations (top 10). A: only included data of mainland China; B: included data of mainland China, Taiwan Province, and Hong Kong.**

**References**

1. Khoo KL, van Acker P, Defesche JC, et al. Low-density lipoprotein receptor gene mutations in a Southeast Asian population with familial hypercholesterolemia. Clin Genet. 2000; 58(2): 98-105.
2. Mak YT, Pang CP, Tomlinson B, et al. Mutations in the low-density lipoprotein receptor gene in Chinese familial hypercholesterolemia patients. Arterioscler Thromb Vasc Biol. 1998; 18(10):1600-1605.
3. Chang JH, Pan JP, Tai DY, et al. Identification and characterization of LDL receptor gene mutations in hyperlipidemic Chinese. J Lipid Res. 2003; 44(10): 1850-1858.
4. Sun XM, Patel DD, Webb JC, et al. Familial hypercholesterolemia in China. Identification of mutations in the LDL-receptor gene that result in a receptor-negative phenotype. Arterioscler Thromb Vasc Biol. 1994; 14(1): 85-94.
5. Hobbs HH, Brown MS, Goldstein JL. Molecular genetics of the LDL receptor gene in familial hypercholesterolemia. Hum Muta. 1992; 1(6): 445-466.
6. Chiou KR, Charng MJ. Common mutations of familial hypercholesterolemia patients in Taiwan: characteristics and implications of migrations from southeast China. Gene. 2012;498(1):100-6.
7. Jiang L, Gao F, Hu LB, et al. Seven-year clinical follow-up of a Chinese homozygous familial hypercholesterolemia child with premature xanthomas and coronary artery disease - A need for early diagnosis and aggressive treatment. Int J Cardiol. 2014;177(1):188-191.
8. Meng X, Lin J, Gao X, et al. Corneal arcus and xanthomas in homozygous familial hypercholesterolemia First report from China. Indian J Ophthalmol. 2013;61(12):770-1.
9. Guan X, Pan XD, Wang CM, et al. Detection of LDL receptor gene mutation in a child patient with familial hypercholesterolemia by whole cDNA sequence analysis. Lin Chuang Jian Yan Za Zhi (Chinese). 2014;32(7):481-484.
10. [Chiou KR](http://www.ncbi.nlm.nih.gov/pubmed/?term=Chiou KR[Author]&cauthor=true&cauthor_uid=20538126), [Charng MJ](http://www.ncbi.nlm.nih.gov/pubmed/?term=Charng MJ[Author]&cauthor=true&cauthor_uid=20538126). Detection of mutations and large rearrangements of the low-density lipoprotein receptor gene in Taiwanesepatients with familial hypercholesterolemia. [Am J Cardiol.](http://www.ncbi.nlm.nih.gov/pubmed/?term=Detection+of+Mutations+and+Large+Rearrangements+of+the+Low-Density+Lipoprotein+Receptor+Gene+in+Taiwanese+Patients+With+Familial+Hypercholesterolemia) 2010;105(12):1752-8.
11. Charng MJ, Chiou KR, Chang HM, et al. Identification and characterization of novel low-density lipoprotein receptor mutations of familial hypercholesterolaemia patients in Taiwan. Eur J Clin Invest. 2006;36(12):866-74.
12. Yang KC, Su YN, Shew JY, et al. LDLR and apoB are major genetic causes of autosomal dominant hypercholesterolemia in a Taiwanese population. J Formos Med Assoc. 2007;106(10):799-807.
13. Wang L, Lin J, Liu S, et al. Mutations in the LDL receptor gene in four Chinese homozygous familialhypercholesterolemia phenotype patients. Nutr Metab Cardiovasc Dis. 2009; 19(6): 391-400.
14. Lin J, Wang LY, Liu S, et al. Identification of a novel splice mutation of low density lipoprotein receptor gene in a Chinese family with familial hypercholesterolemia. Zhonghua Yi Xue Yi Chuan Xue Za Zhi(Chinese). 2004; 21(1): 14-18.
15. Lu Y, Weng Y, Lian CH. Nodular xanthomatosis complicated by familial hypercholesterolemia. Guo Ji Pi Fu Xing Bing Xue Za Zhi(Chinese). 2013,39(2): 86-88
16. Wang YF, Jin HF, Bu DF, et al. Characterization of one novel mutation of the low density lipoprotein receptor gene in patients with familial hypercholesterolemia - one case report. Zhong Guo Shi Yong Nei Ke Za Zhi(Chinese). 2008; 28(6):450-452.
17. Chen K, Mu YM, Wang BA, et al. Two novel mutations 685del 1 and D129G in the low-density lipoprotein receptor gene in a compound heterozygote Chinese family with familial hypercholesterolemia. Metabolism. 2007; 56(5): 636-640.
18. Lin J, Wang LY, Liu S, et al. One LDL-R mutation in a familial hypercholesterolemia patient with xanthomatosis. Zhonghua Pi Fu Ke Za Zhi(Chinese). 2007; 40(12):752-755.
19. Xu SY, Pan XD, Sun LY, et al. Low density lipoprotein receptor gene mutation of amniotic cell in the prenatal diagnosis of familial hypercholesterolemia. Zhonghua Shi Yong Zhen Duan and Zhi Liao Za Zhi(Chinese). 2012; 26(4):344-348.
20. Cao SC, Wang LY, Qin YW, et al. Analysis of low density lipoprotein receptor gene mutations in a Chinese patient with clinically homozygous familial hypercholesterolemia. Chin Med J (Engl). 2003;116(10):1535-8.
21. Zhao WH, Liu YJ, Yang YL, et al. Identification of one novel mutation of the low density lipoprotein receptor gene in Chinese patients with familial hypercholesterolemia. Zhonghua Xin Xue Guan Bing Za Zhi(Chinese). 2002;30(11):658-660.
22. Xie L, Gong QH, Xie ZG, et al. Two novel mutations of the LDL receptor gene associated with familial hypercholesterolemia in a Chinese family. Chin Med J(Engl). 2007; 120(19): 1694-1699.
23. Xiao YM, Yang YC, Zhang H, Zhang T. A case reports of two patients with familial hypercholesterolemia (Chinese). Zhong Guo Xun Zheng Er Ke Za Zhi. 2014;9(5):398-400.
24. Su PY, Wang LY, Lin J, et al. A novel mutation of the LDL receptor gene leading to familial hypercholesterolemia. Eur J Lipid Sci Technol. 2009;111:646-651.
25. Pimstone SN, Sun XM, du Souich C, et al. Phenotypic variation in heterozygous familial hypercholesterolemia: A comparison of Chinese patients with the same or similar mutations in the LDL receptor gene in China or Canada. Arterioscler Thromb Vasc Biol. 1998; 18(2): 309-315.
26. Yao RE, Wang J, Geng J, et al. Identification of LDLR mutations in two Chinese pedigrees with familial hypercholesterolemia. J Pediatr Endocrinol Metab. 2012; 25(7-8): 769-73.
27. Zhao X, Bu L, Qin S, et al. Early development of xanthoma and coronary disease in a young female with homozygous familial hypercholesterolemia. Int J Cardiol. 2014; 176(1):e15-9.
28. Dai YF, Pan XD, Sun LY, et al. Analysis of compound heterozygous mutation of low-density lipoprotein receptor gene in two patients with familial hypercholesterolemia. Zhonghua Jian Yan Yi Xue Za Zhi(Chinese). 2011; 34(5): 454-458.
29. Wang HH, Xu SY, Sun LY, et al. Functional characterization of two low-density lipoprotein receptor gene mutations in two Chinese patients with familial hypercholesterolemia. PLoS One. 2014;9(3):e92703.
30. Liu YR, Tao XM, Chen JZ, et al. Identification of a novel mutation at the point of low density lipoprotein receptor gene from a subject with familial hypercholesterolemia. Sheng Li Xue Bao(Chinese). 2004; 25(5):566-72.
31. Chen JZ, Shang YP, Li JX, et al. gene sequencing research of three familial hypercholesterolemia families. Zhonghua Xin Xue Guan Bing Za Zhi(Chinese). 2002; 30(6):347-50.
32. Pang QF, Wang Y, Xu M, et al. Screening for low-density lipoprotein receptor gene mutations in familial hypercholesterolemia Chinese. Zhonghua Nei Ke Za Zhi (Chinese). 2004;43(9):665-8.
33. Wen L, Peng WH, Han XG, et al. Mutation analysis of low density lipoprotein receptor gene in patient and his family familial hypercholesterolemia. Lin Chuang Xin Xue Guan Bing Za Zhi (Chinese). 2011;27(6):452-456.
34. Wang D, Wu B, Li Y, et al. A Chinese homozygote of familial hyperchoesterolemia: identification of a novel C263R mutation in the LDL receptor gene. J. Hum. Genet. 2001;46: 152–154.
35. Guan XY, Li MF, Fan LM, Chen Q. Analysis of low density lipoprotein receptor function and gene mutation in familial hypercholesterolemic patients. Zhonghua Yi Xue Yi Chuan Xue Za Zhi (Chinese). 2003;20(2):138-42.
36. Chiu CY, Wu YC, Jenq SF, et al. Mutations in low-density lipoprotein receptor gene as a cause of hypercholesterolemia in Taiwan. Metabolism. 2005;54(8):1082-6.
37. Zhu DM, Chen ZJ, Chen BS, et al. A study on point mutations of lipoprotein receptor gene in patients with hypercholestemia. Zhong Guo Xun Huan Za Zhi (Chinese). 2001;16(3):180-182.
38. Jia F, Wu CF, Lu GP. Analysis of pathogenic gene mutation in one homozygous familial hypercholesterolemia phenotype patient. Guo Ji Xin Xue Guan Bing Za Zhi(Chinese). 2009;36(6):397-398.
39. Wu WF, Sun LY, Pan XD, et al. Use of targeted exome sequencing in genetic diagnosis of Chinese familial hypercholesterolemia. PLoS One. 2014;9(4):e94697.
40. Liang DG, Wang ZX, Gao YX, et al. A novel mutation in a young patient underwent coronary arterty bypass grafting with familial hypercholesterolemia. Exp Clin Cardiol. 2014;20(1):1516-1523.
41. Wang HH, Wang CM, Xu SY, et al. Functional analysis on novel mutants of low density lipoprotein receptor gene in a familial hypercholesterolemia patient. Lin Chuang Jian Yan Za Zhi(Chinese). 2014;32(5):324-328.
42. Lai H, Fong JB, Wang T, et al. A genetic and clinical study in a family with familial hypercholesterolemia. Zhonghua Nei Ke Za Zhi(Chinese). 2011;50(2):120-123.
43. Lin J, Wang LY, Liu S, et al. Functional analysis of low-density lipoprotein receptor in homozygous familial hypercholesterolemia patients with novel 1439 C-->T mutation of low-density lipoprotein receptor gene. Chin Med J (Engl). 2008;121(9):776-81.
44. Liu S, Wang LY, Lin J, et al. Detection of W462X mutation in low density lipoprotein receptor gene of a familial hypercholesterolemia patient and its clinical significance. Shi Yong Er Ke Lin Chuang Za Zhi (Chinese). 2009;24(1):18-23.
45. Cheng XH, Zheng F, Zhou X, et al. Mutation screening and functional analysis of low density lipoprotein receptor in a familial hypercholesterolemia family Zhonghua Yi Xue Yi Chuan Xue Za Zhi(Chinese).. 2008;25(1):55-8.
46. Cheng XH, Ding JF, Zheng F, Zhou X, Xiong CL. Two mutations in LDLR gene were found in two Chinese families with familial hypercholesterolemia. Mol Biol Rep. 2009;36(8): 2053-2057.
47. Wang X, Lin J, Pan XD, et al. Analysis of low density lipoprotein receptor gene mutation in a child and his family with familial hypercholesterolemia. Shi Yong Er Ke Lin Chuang Za Zhi (Chinese). 2010;25(2):125-128.
48. Wu H, Xie F, Lang XL, et al. Mutation analysis of low-density lipoprotein receptor gene in 3 patients with familial hypercholesterolemia in two generations. Di Er Jun Yi Da Xue Xue Bao(Chinese). 2012;33(4):445-448.
49. Sun LY, Pan XD, Su PY, et al. Analysis of LDL receptor exon 13 mutation in patient with familial hypercholesterolemia (Chinese). Xian Dai Sheng Wu Yi Xue Jin Zhan. 2010;10(13):2451-2454.
50. Sun P, Guo DP, Li XY, et al. Analysis of gene mutation in the low density lipoprotein receptor genes of one familial hypercholesterolemia genealogy (Chinese). Zhong Guo Dong Mai Ying Hua Za Zhi. 2004;12(5):577-580.
51. Pan XD, Su PY, Wang LY, et al. Analysis of apoliporotein B gene in a proband with familial hypercholesterolemia (Chinese). Lin Chuang Jian Yan Za Zhi. 2010;28(3):179-181.
52. Faiz F, Hu M, Hooper AJ, et al. Molecular characterization of a Chinese woman homozygous for the familial hypercholesterolemia LDLR c.1474G>A (p.Asp492Asn) mutation. Clin Lipidol. 2014;9(2):163-170.
53. Zhou YL, Zhao YJ, Cui B, et al. Genetic analysis in the patients with familial hypercholesterolemia (Chinese). Zhen Duan Xue Li Lun Yu Shi Jian. 2005;4(6):473-476.
54. Chen LW, Yang M, Lin J, Wang LY. Novel mutation of low density lipoprotein receptor gene associated with familial hypercholesterolemia. Zhong Guo Yi Shi Jin Xiu Za Zhi(Chinese). 2010;33(22):1-4.
55. Zhou WJ, Wang X, Jin M. Analysis low density lipoprotein receptor mutation in a familial hypercholesterolemia family. Shi Yong Yi Xue Za Zhi(Chinese). 2010;26(22):4134-4137.
56. Xu YJ, Wang LY, Lin J, et al. Analysis of LDL receptor function and gene mutations in familial homozgous hypercholesterolemia (Chinese). Lin Chuang Xin Xue Guan Bing Za Zhi. 2008;24(5):350-354
57. Sun LY, Pan XD, Wang X, et al. Study on the mutation of low density lipoprotein receptor gene in familial hypercholesterolemia patients with premature coronary heart disease (Chinese). Zhong Guo Yi Yao. 2011;6(8):900-901.
58. Sun LY, Pan XD, Su PY, et al. Gene mutation analysis of a child with familial hypercholesterolemia and his family (Chinese). Shi Yong Er Ke Lin Chuang Za Zhi. 2010;25(13):968-973.
59. Cheng XY, Cheng XH, Zhang Y, et al. The phenotypes of a hypercholesterolemia family with low density lipoprotein receptor exon 13 A606T mutation (Chinese). Zhonghua Nei Ke Za Zhi. 2012;51(9):680-682.
60. Wang DQ, Li Y, Mu Y, et al. Analysis of the low density lipoprotein receptor(LDLR)gene mutations in children with familial hypercholesterolemia (Chinese). Zhonghua Er Ke Za Zhi. 2001;39(3):134-137.
61. Pan XD, Su PY, Wang LY, et al. Analysis of apolipoprotein B gene in a proband with familial hypercholesterolemia (Chinese). Lin Chuang Jian Yan Za Zhi. 2010;28(3):179-181.
62. Xia JH, Wang LY, Lin J, et al. Mutation analysis of low density lipoprotein receptor gene in a child with familial hypercholesterolemia and coronary heart disease (Chinese). Shi Yong Er Ke Lin Chuang Za Zhi. 2008;23(1):25-27.
63. Li XD, Wang JY, Hu WC. A new mutation in patients with familial hypercholesterolemia in low density lipoprotein receptor gene (Chinese). Zhonghua Yi Xue Yi Chuan Xue Za Zhi. 2001;18(6):491-492.
64. Chiou KR, Charng MJ, Chang HM. Array-based resequencing for mutations causing familial hypercholesterolemia. Atherosclerosis. 2011;216(2):383-389.
65. Yang KC, Su YN, Shew JY, et al. LDLR and ApoB are major genetic causes of autosomal dominant hypercholesterolemia in a Taiwanese population. J Formos Med Assoc. 2007;106(10):799-807.
66. Feng Z, Di JF, Zeng S, Feng JS. Rapid cUagnosis of familial defective apolipoprotein B-100 R3SOOW by mutagenic PCR primers (Chinese). Lin Nan Xin Xue Guan Bing Za Zhi. 2002;8(1):10-12.
67. Feng JS, Zhou Y, Yu RM, Lin CL. Screening for familial defective apolipoprotein B-100 in hyperlipidemic Chinese:Identification of a case of R 3500W mutation (Chinese). Zhong Hua Xin Xue Guan Bing Za Zhi. 2000;28(6):449-451.
68. Teng YN, Pan JP, Chou SC, Tai DY, Lee-Chen GJ. Familial defective apolipoprotein B-100: detection and haplotype analysis of the Arg(3500)-->Gln mutation in hyperlipidemic Chinese. Atherosclerosis. 2000;152(2):385-90.
69. Huang JJ, Zhou X, Chen F, Ha DW. Screening of Familial Defective Apol ipoprotein B-100 (Chinese). Zhong Hua Xun Huan Za Zhi. 1998;13(5):284-286.
70. Tai DY, Pan JP, Lee-Chen GJ. Identification and haplotype analysis of apolipoprotein B-100 Arg3500-->Trp mutation in hyperlipidemic Chinese. Clin Chem. 1998;44(8):1659-65.
71. Wu JH, Lee YT, Hsu HC, et al. Further characterization of apolipoprotein B genetic variations in Taiwanese. Hum Biol. 2001;73(3):451-60.
72. Abdel-Wareth LO, Pimstone SN, Lagarde JP, et al. Familial defective apolipoprotein B-100 in hypercholesterolemic Chinese Canadians: identification of a unique haplotype of the apolipoprotein B-100 allele. Atherosclerosis. 1997;135(2):181-5.
73. Song GY, Zhang MM, Sun HJ, et al. Research of Gene Mutation of Proprotein Convertase Subtilisin /Kexin 9 in Hypercholesterolemia (Chinese). Zhong Guo Dong Mai Ying Hua Za Zhi. 2012;20(8):731-735.
74. Lin J, Wang LY, Liu S, et al. A novel mutation in proprotein convertase subtilisin/kexin type 9 gene leads to familial hypercholesterolemia in a Chinese family. Chin Med J (Engl). 2010;123(9):1133-8.
